# Corneal Alterations during Combined Therapy with Cyclodextrin/Allopregnanolone and Miglustat in a Knock-Out Mouse Model of NPC1 Disease

**DOI:** 10.1371/journal.pone.0028418

**Published:** 2011-12-06

**Authors:** Marine Hovakimyan, Jana Petersen, Fabian Maass, Maria Reichard, Martin Witt, Jan Lukas, Oliver Stachs, Rudolf Guthoff, Arndt Rolfs, Andreas Wree

**Affiliations:** 1 Department of Ophthalmology, University of Rostock, Rostock, Germany; 2 Department of Anatomy, University of Rostock, Rostock, Germany; 3 Albrecht-Kossel-Institute for Neuroregeneration, University of Rostock, Rostock, Germany; The University of Hong Kong, Hong Kong

## Abstract

**Background:**

Niemann Pick disease type C1 is a neurodegenerative disease caused by mutations in the NPC1 gene, which result in accumulation of unesterified cholesterol and glycosphingolipids in the endosomal-lysosomal system as well as limiting membranes. We have previously shown the corneal involvement in NPC1 pathology in form of intracellular inclusions in epithelial cells and keratocytes. The purpose of the present study was to clarify if these inclusions regress during combined substrate reduction- and by-product therapy (SRT and BPT).

**Methodology/Principal Findings:**

Starting at postnatal day 7 (P7) and thereafter, NPC1 knock-out mice (NPC1^−/−^) and wild type controls (NPC1^+/+^) were injected with cyclodextrin/allopregnanolone weekly. Additionally, a daily miglustat injection started at P10 until P23. Starting at P23 the mice were fed powdered chow with daily addition of miglustat. The sham group was injected with 0.9% NaCl at P7, thereafter daily starting at P10 until P23, and fed powdered chow starting at P23. For corneal examination, in vivo confocal laser-scanning microscopy (CLSM) was performed one day before experiment was terminated. Excised corneas were harvested for lipid analysis (HPLC/MS) and electron microscopy.

In vivo CLSM demonstrated a regression of hyperreflective inclusions in all treated NPC1^−/−^mice. The findings varied between individual mice, demonstrating a regression, ranging from complete absence to pronounced depositions. The reflectivity of inclusions, however, was significantly lower when compared to untreated and sham-injected NPC1^−/−^ mice. These confocal findings were confirmed by lipid analysis and electron microscopy. Another important CLSM finding revealed a distinct increase of mature dendritic cell number in corneas of all treated mice (NPC1^−/−^ and NPC1^+/+^), including sham-treated ones.

**Conclusions/Significance:**

The combined substrate reduction- and by-product therapy revealed beneficial effects on the cornea. In vivo CLSM is a non-invasive tool to monitor disease progression and treatment effects in NPC1 disorder.

## Introduction

Lysosomal storage diseases (LSDs) are a form of metabolic disorder caused by inherited deficiencies of specific lysosomal enzymes, which lead to the accumulation of nonmetabolized macromolecules [1]. The frequency of LSDs as a group varies among populations from 7 to 25 per 100.000 [2].

Niemann Pick disease type C1 is a LSD of autosomal recessive inheritance, caused by mutations in the NPC1 gene that encodes for a large transmembrane protein [3]. In Western Europe, the birth incidence of NPC1 has been estimated to be 1 in 150.000 [4]. Cells harbouring mutations in NPC1 gene accumulate low-density lipoprotein (LDL)-derived cholesterol in late endosomes/lysosomes and exhibit defects in lipid trafficking and storage [5], [6]. Affected patients develop ataxia, motor dysfunction and organomegaly [7], [8]. The neuropathological features are characterized by progressive loss of Purkinje cells in the cerebellum, and neurons in the basal ganglia and brain stem [9], [10]. Also, cytoskeletal changes have been demonstrated in neurons in form of neurofibrillary tangles, consisting of hyperphosphorylated tau protein [11]. The initial clinical manifestations of NPC1 disease vary strongly, being neurological, pulmonary or hepatic in nature [12]. The lifespan of patients varies between a few days until over 60 years of age - the majority of cases, however, die between 10 and 25 years of age [13].

One possibility to alleviate the severity of disease could be blocking the intestinal absorption of cholesterol with Ezetimibe [14] or inhibition of protein hyperphosphorylation. Blocking of cyclin-dependent kinases reveal a strong inhibitory effect on protein phosphorylation, being favourable for neural cell survival and thus improving locomotor defects in NPC1 knock-out (NPC1^−/−^) mice [15]. However, the inhibition of extracellular signal regulated kinases did not alter neurological indices of NPC1 disease in this mouse model [16].

A very promising approach for the treatment of NPC1 is the substrate reduction therapy (SRT) with the blood-brain barrier crossing small molecule miglustat (N-butyldeoxynojirimycin), which has been reported to reduce lipid accumulation in NPC1^−/−^ mice, thus leading to delay in onset of clinical signs and increasing lifespan [17]. Administration of miglustat has normalized lipid trafficking and improved clinical signs in human patients, both children and adults [18]–[22].

The second therapeutic approach, so called by-product therapy (BPT) utilizes the neurosteroid allopregnanolone, which is deficient in NPC1^−/−^ mice [23]. Allopregnanolone, dissolved in 2-hydroxypropyl-ß-cyclodextrin (CD), led to delay in clinical onset and decrease of ganglioside deposition [24]. It could be shown that combination of both approaches (miglustat and cyclodextrin/allopregnanolone) had a significant synergic effect in ameliorating disease progression [25]. Very interestingly, the same study demonstrated that administration of vehicle cyclodextrin even at low concentrations had a greater therapeutic effect in NPC1^−/−^ mice than did the administration of allopregnanolone alone.

Recently, we have reported on the visualization of hyperreflective inclusions in corneal epithelial cells in NPC1 deficient mice by *in vivo* confocal laser-scanning microscopy (CLSM), a non-invasive technique [26]. The present study was designed for investigation of NPC1^−/−^ mice corneas after combined SRT and BRT, including cyclodextrin, allopregnanolone and miglustat (Cyclo/ALLO/miglustat). We hypothesized that treatment effects could be monitored by this *in vivo* imaging possibility, thus, giving researchers and clinicians an additional tool for monitoring disease lapse and treatment efficiency among neurological and biochemical examinations.

## Results

### In vivo CLSM

**Figure 1:**
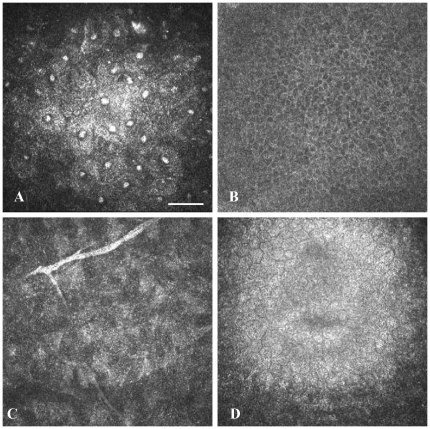
In vivo CLSM of NPC1^+/+^ mouse cornea. (A) - superficial epithelial cells with hyperreflective nuclei and dark cytoplasm. (B) - basal epithelial cells with polygonal morphology and bright cell borders. (C) - corneal stroma with dark ground substance and hyperreflective nerves. (D) - hexagonal endothelial cells with highly reflective cytoplasm and dark cell borders. Bar: 50 µm.

In vivo CLSM allowed visualization of all corneal layers at the cellular level (Fig. 1).

The most superficial cells of a normal cornea (NPC1^+/+^ mice) were visualised as polygonal structures with bright cytoplasm and hyperreflective nuclei (Fig. 1A). The smaller basal cells were characterized by bright cell borders and a dark cytoplasm, without distinguishable nuclei (Fig. 1B). The corneal stroma demonstrated a dark background (extracellular matrix), reflective interconnected stellate structures, corresponding to keratocyte cell bodies and hyperreflective stromal nerves (Fig. 1C). The endothelial monolayer consisted of hexagonal cells with bright cytoplasm, displaying minimal variations in size and morphology (Fig. 1D).

**Figure 2:**
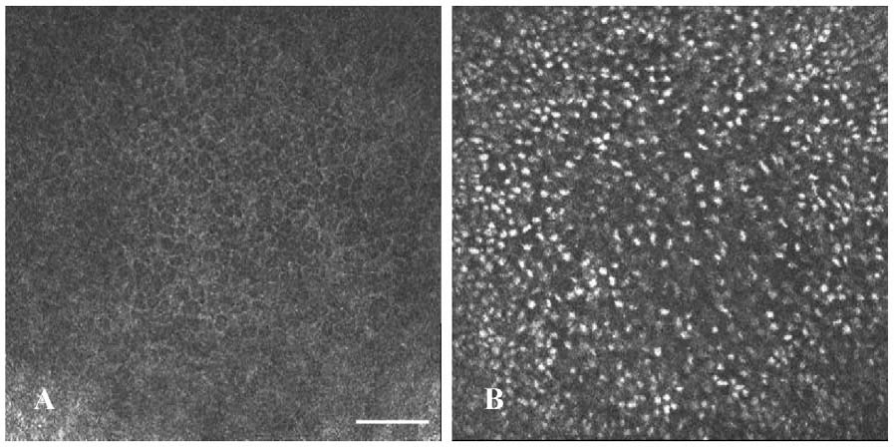
In vivo CLSM of basal epithelial cells in NPC1^+/+^ and NPC1^−/−^ mouse cornea. Compared to the normal epithelium of NPC1^+/+^ mice (A), the basal epithelial cells of NPC1^−/−^ mice revealed no visible cell borders and excessive hyperreflective inclusions (B). Bar: 50 µm.

Figure 2 demonstrates structural changes affecting basal cells in NPC1 disease. When compared to the normal morphology of basal epithelial cells seen in NPC1^+/+^ mice (Fig. 2A), the basal cells in NPC1^−/−^ cells showed no distinguishable borders and cytoplasm, but were identified as units with hyperreflective content (Fig. 2B).

**Figure 3:**
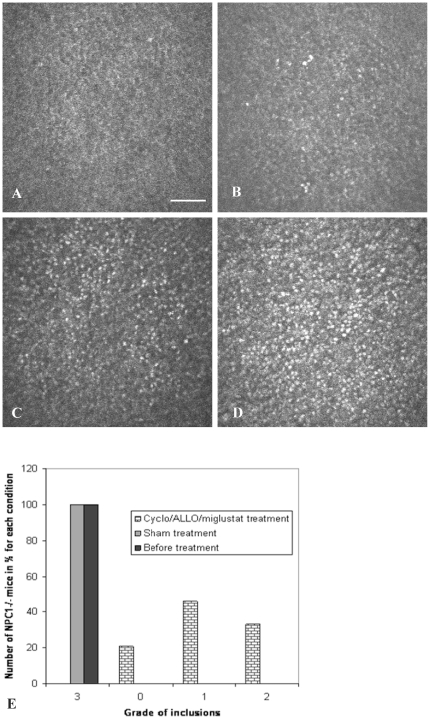
Grading system of corneal epithelial inclusions following in vivo CLSM in Cyclo/ALLO/miglustat-treated NPC1^−/−^ mice. (A) - almost no inclusions, grade 0. (B) - isolated hyperreflective structures in the basal cell layer, less than 25% of cells are affected-grade 1. (C)- up to 50% of cells content hyperreflective inclusions-grade 2. (D) - for comparison, the cornea of a sham-treated NPC1^−/−^ mouse - more than 75% of cells are affected, grade 3. Bar: 50 µm. (E) - A graph, representing the number of NPC1^−/−^ mice with corneal inclusions of different grades (0–3) before and after treatment.

*In vivo* CLSM revealed regression of hyperreflective inclusions in epithelial cells after Cyclo/ALLO/miglustat treatment. Importantly, the corneal response to the treatment varied strongly between individual mice. We therefore classified the inclusions in a grading system. From out of 14 Cyclo/ALLO/miglustat-treated NPC1^−/−^ mice 3 almost lacked inclusions (grade 0, Fig. 3A). Among residual 11 NPC1^−/−^ mice, grade 1 (up to 25% of cells affected) occurred in 5 (Fig. 3B) and grade 2 (up to 50% of cells affected) in 6 mice (Fig. 3C). In sham-treated NPC1^−/−^ corneas the depositions of very high intensity affected almost every single cell (grade 3, Fig. 3D). To date, the grade 3 was never observed in Cyclo/ALLO/miglustat-treated NPC1^−/−^ mice. These findings are summarized in a graph (Fig. 3E), which clearly shows the percentage of NPC1^−/−^ mice involved in a grading system from 0 to 3, before and after treatment.

**Figure 4:**
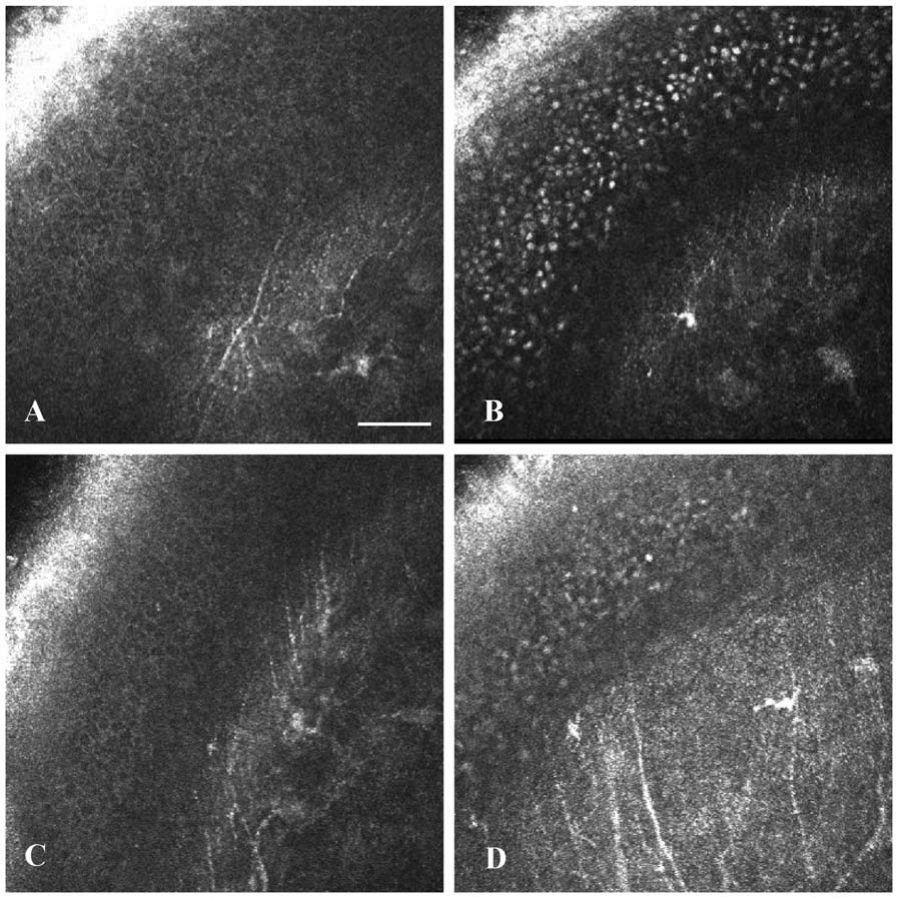
In vivo CLSM of cornea, oblique section. (A) - normal cell morphology in NPC1^+/+^ cornea. (B) - inclusions in intermediate and basal cells in NPC1^−/−^ cornea. NPC1^−/−^ corneas after treatment: (C) - almost no inclusions after Cyclo/ALLO/miglustat therapy, (D) - severe inclusions with low intensity, after Cyclo/ALLO/miglustat therapy. Bar: 50 µm.

These observations become more obvious in oblique sections of the epithelium (Fig. 4). The oblique section shows clearly hyperreflective inclusions in intermediate and basal cells in NPC1^−/−^ corneas (Fig. 4B). The corneas of some Cyclo/ALLO/miglustat-treated NPC1^−/−^ mice revealed almost no inclusions (Fig. 4C) when compared to controls (Fig. 4A), whereas corneas from other treated NPC1^−/−^ mice revealed more pronounced inclusions (Fig. 4D, grade 2), whose intensity was, however, much lower when compared to untreated ones.

**Figure 5:**
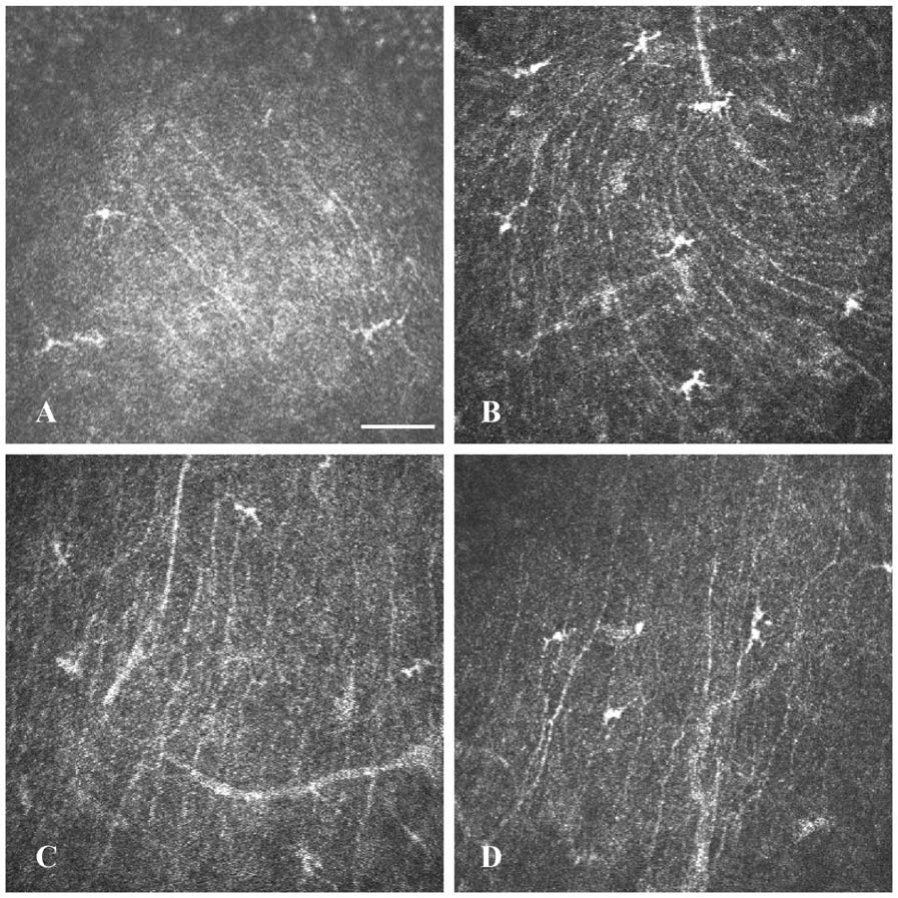
Corneal Dendritic cells in in vivo CLSM. (A) **-** Dendritic cells in the cornea of a NPC1^−/−^ mouse before treatment. Massive accumulation of DCs in the corneas of NPC1^−/−^ (B) and NPC1^+/+^ mice (C) after Cyclo/ALLO/miglustat-treatment. Also sham-treated mice revealed a dense population of DCs (D). Bar represents 50 µm.

**Figure 6:**
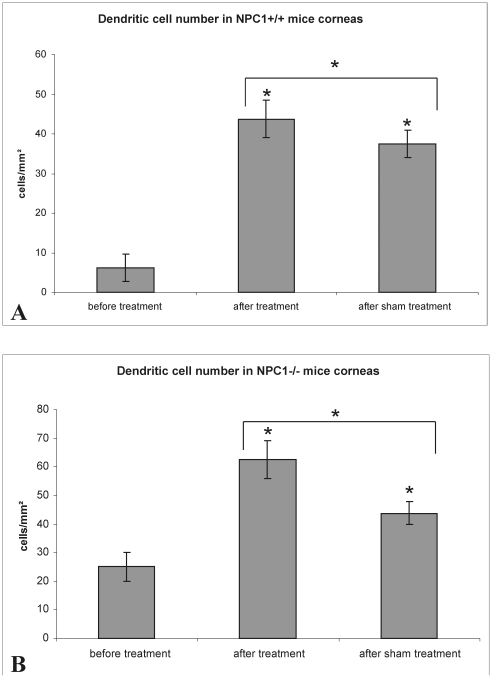
Number of dendritic cells before and after treatment in NPC1^+/+^ and NPC1^−/−^ mouse corneas. (A) **-** the number of dendritic cells in NPC1^+/+^ corneas increased after Cyclo/ALLO/miglustat and sham treatment. In both cases the increase was statistically significant (p<0.0001). (B) - also the corneas of NPC1^−/−^ mice revealed a statistically significant increase in dendritic cell number both after Cyclo/ALLO/miglustat and sham treatment (p<0.0001).

Normally, there are only very few dendritic cells (DCs) observed in the central mouse cornea. They become obvious only after careful examination of the entire central part of the cornea. A considerable increase in DC number was noted in NPC1^−/−^ corneas (Fig. 5A). After Cyclo/ALLO/miglustat treatment, in both groups (NPC1^−/−^ and NPC1^+/+^) *in vivo* CLSM revealed numerous mature DCs with processes arranged in a meshwork (Fig. 5B and C). Interestingly, also the sham-treated group revealed very pronounced DCs (Fig. 5D). The number of DCs was quantified in all groups (Table 1). In the NPC1^+/+^ group (Fig. 6A) the number of DCs increased significantly from 9±3 to 45±5 cells/mm^2^ after Cyclo/ALLO/miglustat therapy (p<0.0001). Also the sham-treated NPC1^+/+^ mice revealed an increase to 34±3 cells/mm^2^, which was statistically significant when compared to pre-treatment value (p<0.0001). Similarly, the NPC1^−/−^ corneas (Fig. 6B) demonstrated a significant increase of DC number from 29±5 cells/mm^2^ to 72±6 cells/mm^2^ (p<0.0001) after Cyclo/ALLO/miglustat therapy and to 50±4 cells/mm^2^ (p<0.0001) after sham treatment. Notably, the increase of DC number was more pronounced after Cyclo/ALLO/miglustat therapy: the comparison of both forms of therapy by mean of DC number revealed a significant difference, both for NPC1^+/+^ (p = 0.00068) and NPC1^−/−^ (p = 0.000018) mice corneas.

**Table 1:** Dendritic cell number in the central cornea of NPC1^+/+^ and NPC1^−/−^ mice before and after treatment (Cyclo/ALLO/miglustat or sham).

	Number of DCs (cells/mm^2^)		
	Before treatment	After Cyclo/ALLO/miglustat-traetment	After sham treatment
NPC1^+/+^	9±3	45±5	34±3
NPC1^−/−^	29±5	72±6	50±4

### High Performance Liquid Chromatography/Mass spectroscopy (HPLC/MS)

**Figure 7:**
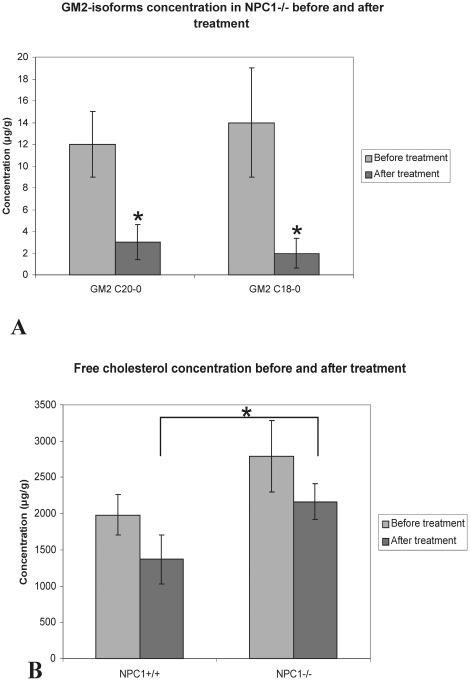
Levels of GM2-isoforms and free cholesterol before and after Cyclo/ALLO/miglustat-treatment. (A) - lipid analysis showed a decrease of free cholesterol level after Cyclo/ALLO/miglustat-treatment, both in NPC1^+/+^ and NPC1^−/−^ mice (statistically not significant). (B) - levels of both isoforms of GM2 revealed a statistically significant decrease in NPC1^−/−^ after Cyclo/ALLO/miglustat-treatment, when compared to controls. For both, C20-0 and C18-0 the differences were statistically significant.

The levels of two different isoforms of disialotetrahexosylganglioside 2-GM2 (GM2 C20-0 and GM2 C18-0) were negligible (lower than level of quantification-LLOQ, less than 4 µg/g) in NPC1^+/+^ mice corneas (n = 4). Therefore it was not possible to evaluate the impact of Cyclo/ALLO/miglustat therapy on GM2 level in NPC1^+/+^ corneas. In contrast, the NPC1^−/−^ corneas (n = 4) revealed a highly increased level of GM2 isoforms before treatment, reaching 12 and 14 µg per g tissue for GM2 C20-0 and GM2 C18, respectively. After Cyclo/ALLO/miglustat therapy the HPLC/MS analysis demonstrated that the GM2 C20-0 and GM2 C18-0 levels were dramatically reduced in Cyclo/ALLO/miglustat-treated NPC1^−/−^ mice corneas, reaching 3 and 2 µg per g tissue, respectively (Fig. 7A). For both, C20-0 and C18-0 this reduction reached significance (p = 0.02).

The corneas of Cyclo/ALLO/miglustat-treated NPC1^+/+^ and NPC1^−/−^ mice revealed also a reduction in free cholesterol level in both groups (Fig. 7B). The level of cholesterol was reduced from 1984±164 µg/g (before treatment) to 1367±493 µg/g (after Cyclo/ALLO/miglustat treatment) in NPC1^+/+^ mice and from 2792±599 µg/g (before treatment) to 2165±248 µg/g (after Cyclo/ALLO/miglustat treatment) in NPC1^−/−^ treated ones. This decrease was, however, not significant for both, NPC1^+/+^ and NPC1^−/−^ mice corneas (p = 0.0564 for NPC1^+/+^ group and p = 0.083 for NPC1^−/−^ group).

The comparison of free cholesterol level between NPC1^+/+^ and NPC1^−/−^ group revealed a statistically not significant increase in NPC1^−/−^ group before treatment. After Cyclo/ALLO/miglustat therapy the difference in free cholesterol level was still relatively small between NPC1^+/+^ and NPC1^−/−^, reaching, however, significance (p = 0.02).

As mentioned above, the corneas for lipid analysis were harvested from randomly chosen mice. To verify the treatment effects, a comparison between HPLC data and *in vivo* CLSM findings was performed.

By comparison of absolute values of HPLC/MS (Table 2) with *in vivo* CLSM findings a very interesting observation was made: those mice having intracellular deposits of grade 0 or 1 in *in vivo* CLSM also had negligible GM2 values in HPLC/MS (LLOQ), whereas the mice with more pronounced accumulations in CLSM demonstrated detectable amounts of both isoforms of GM2, which were, however, strongly decreased when compared to pre-treatment values.

**Table 2:** Comparison of HPLC/MS data with in vivo CLSM findings.

Mouse number	GM2 C20-0	GM2C18-0	*In vivo* CLSM
1062(NPC1^−/−^)	LLOQ	LLOQ	Grade 0
1068(NPC1^−/−^)	5,15	4,37	Grade 2
1061(NPC1^−/−^)	6,03	5,17	Grade 2
1087(NPC1^−/−^)	LLOQ	LLOQ	Grade 1

### Electron microscopy

**Figure 8:**
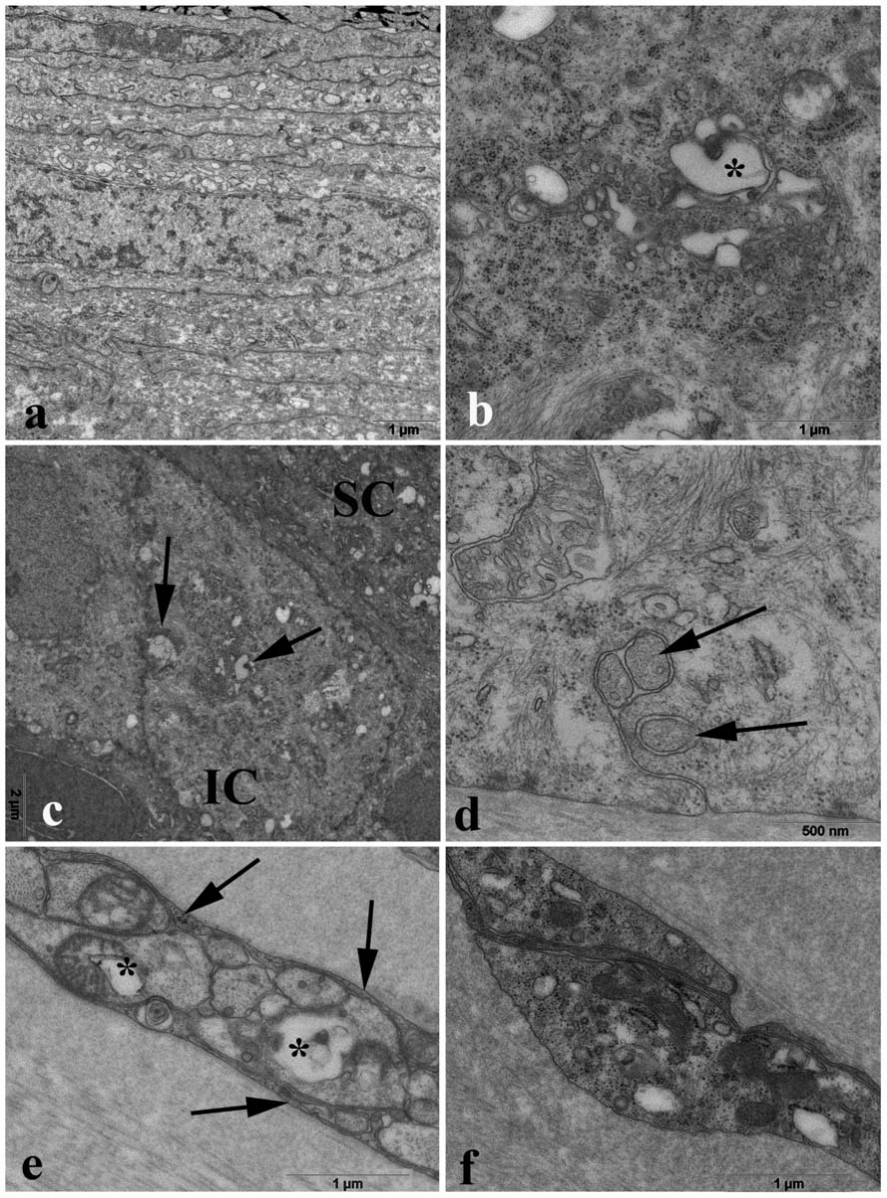
Electron microscopical depictions of treated NPC1 mice. (A) - superficial epithelial cells of a treated NPC1^−/−^ mouse without pathological inclusions. (B) - regular cytoplasm of an intermediate epithelial cell. Rarely, somewhat enlarged profiles of Golgi compartments can be found (asterisk, detail from C). (C) - intermediate epithelial cell with some enlarged membrane-bound compartments, presumably derived from Golgi cisterns. (D) - basal layer of a regular corneal epithelium of NPC1^+/+^ mouse. The arrows indicate axons wrapped by basal epithelial cells close to the Bowman membrane. (E) - unmyelinated nerve fibers in the corneal stroma. Some of the fibers contain enlarged electron-light compartments with small electron-dense corpuscles similar to that seen in B. Other cell organelles (mitochondria, neurotubules, and vesicles) are regular. Schwann cell processes (arrows) do not contain pathological inclusions. (F) – keratocytes of the stroma show a regular aspect.

In one Cyclo/ALLO/miglustat-treated NPC1^−/−^ mouse cornea that showed only sporadic CLSM reflectivity (mouse 1089), we did not observe myelin-like material compared to that found in non-treated NPC1^−/−^ corneas. The superficial epithelial cells did not contain any pathological intracellular inclusions (Fig. 8A). Occasionally, some enlarged electron-lucent compartments (probably Golgi cisterns) were seen in intermediate epithelial cells (Fig. 8B, C) rather than in basal cells (Fig. 8D). Unmyelinated nerve fibers in several areas and levels of basal epithelium and corneal stroma were largely void of inclusions (Fig. 8D, E). Also, keratocytes of the stroma did not show any deposits after combined drug therapy (Fig. 8F).

## Discussion

The NPC1 phenotype observed in BALB/c npc^nih^ mice results from a mutation in NPC1 gene-the same gene which is responsible for NPC1 disease in humans [27]. These mice exhibit a deficiency of NPC1 protein with subcellular consequences, including disturbances in sterol metabolism and trafficking [28]. Pathological features in NPC1^−/−^ mice resemble those observed in late infantile NPC1 disease in humans, exhibiting progressive neurodegeneration, hepatosplenomegaly and ataxia [29]. The mice revealed excessive lipid deposition in different tissues, causing permanent cellular damage, particularly in the nervous system, liver, spleen, lungs and bone marrow [27]. Cholesterol and glycosphingolipids such as GM2 and neutral glycolipids have been reported to be predominantly accumulated in the NPC1^−/−^ mouse [30], [31]. Consistently, we could show accumulation of GM2 in the cornea of NPC1^−/−^ mice [26]. Interestingly, no unesterified cholesterol storage could be found in the cornea, whereas the retina of the same mice revealed excessive accumulation of free cholesterol [32], suggesting different storage patterns even within different tissues.

The ophthalmological examination has been reported to be of particular interest in NPC1 disease, because abnormal saccadic eye movements (SEM) are one of the earliest neurological signs of disease onset [33]. The deficit in SEM occurs both in vertical and horizontal plane. Another sign of NPC1 disease addressing to ocular involvement is the macular cherry red spot, which is one of most important symptoms in the diagnosis of almost all storage diseases [34], [35]. The corneal involvement in NPC1 disease has been only seldom reported, and even these rare data are inconsistent, reporting in one case on corneal inclusions [36] and in another case on normal corneal morphology without any abnormalities [37], even though the same techniques (histology and electron microscopy) were used in both studies. In our previous work we demonstrated for the first time corneal inclusions in NPC1^−/−^ mice by using *in vivo* CLSM. The *in vivo* CLSM findings were confirmed by lipid analysis and electron microscopy [26]. The *in vivo* CLSM is a relatively new but very promising approach for corneal examination in LSDs. This technique has a big advantage to be non-invasive, thus enabling dynamic studies in the same individuals over time. The corneal involvement has been demonstrated by using *in vivo* CLSM in patients with other LSDs, like Fabry disease, Tangier disease and cystinosis [38]–[40].

On the basis of our *in vivo* CLSM finding about corneal involvement in NPC1 disease, we performed the present study to further clarify whether the regression of cellular inclusions could be achieved by combined Cyclo/ALLO/miglustat therapy. This combined approach has been shown to have alleviating effects in NPC1^−/−^ mice [25]. The treated NPC1^−/−^ mice revealed delayed onset of ataxic gait and tremor, increased lifespan and significant reduction of cholesterol and ganglioside accumulation. In good agreement with Davidson et al. (2009), [25], we visualised the regression of accumulations in corneal epithelium of NPC1^−/−^ mice. Notably, the *in vivo* CLSM findings varied between individual mice, revealing an absolute remission of deposits in some animals, and still pronounced accumulations in others. Overall, however, the number of affected cells and intensity of inclusions were significantly reduced in corneas of Cyclo/ALLO/miglustat-treated NPC1^−/−^ mice when compared to untreated or sham-treated NPC1^−/−^ mice corneas. These findings were confirmed by lipid analysis, which demonstrated a significant reduction of both examined isoforms of GM2 and a slight reduction of cholesterol concentration. These findings could be further confirmed by using electron microscopy, which also revealed a relatively normal corneal morphology, without apparent intracellular inclusions. It should be pointed out, that the free cholesterol concentration differed not significantly between both groups before treatment. This difference became, however, statistically significant after Cyclo/ALLO/miglustat treatment. We proposed that this might have been caused by a more pronounced decrease of free cholesterol concentration in NPC1^+/+^ mice corneas (31% from initial values in NPC1^+/+^ versus 22% in NPC1^−/−^ mice corneas). Accordingly, we would also expect a more pronounced decrease of GM2 levels in NPC1^+/+^ mice corneas. We could not prove this, however, because the levels of both isoforms of GM2 were lower than the limit of quantification in NPC1^+/+^ mice corneas. The main scope of our study was, however, the *in vivo* examination of hyperreflective inclusions following Cyclo/ALLO/miglustat treatment. Biochemical analysis and electron microscopy were performed for confirmation of *in vivo* findings, and only on a limited number of animals. Future biochemical studies with sufficient number of mice (n = 6 or 8) should be performed to clarify whether (and why) the corneas of wild type and NPC1^−/−^ mice respond differently to Cyclo/ALLO/miglustat treatment.

Another important finding was the increase of dendritic cell (DC) number in both treated groups. To our knowledge, one possible explanation could be the mild allergic inflammation of corneal surface without functional specificity. Alternatively, this reaction could be caused by powdered chow which all mice were fed starting at P23 until termination of experiments. The powdered chow could have induced mechanical stress in the cornea, leading probably to the migration of mature DCs from the periphery to the central cornea, thus, resulting in the increase of DCs in the central cornea. This hypothesis could be supported by the fact that sham-treated mice, which got their chow in powdered form, also demonstrated an increase of DCs number like Cyclo/ALLO/miglustat-treated ones. Nevertheless, an additional statistical analysis between sham-treated and Cyclo/ALLO/miglustat-treated animals showed a significant difference between two treatment options, both in NPC1^+/+^ and NPC1^−/−^ groups. Thus, we are inclined to believe that apart from a mild stress, caused by powdered chow, Cyclo/ALLO/miglustat treatment itself contributed, at least partially, to the increase of DCs.

It is widely known that one of the most pronounced side effects of miglustat treatment in Gaucher and NPC1 patients is the peripheral neuropathy [41]. Immune mechanisms have been proposed to play an important role in the development of peripheral neuropathy in the cornea [42]. Thus, the increased number of DCs can be considered as a part of mechanisms suggesting immune-mediated contribution to the neuropathy.

Importantly, the NPC1^−/−^ mice had already had a high number of DCs before treatment, when compared to NPC1^+/+^, and also after the treatment they revealed the most pronounced increase of DC number. This pronounced increase can be attributed to some extent to be part of natural progression of disease. In the future, *in vivo* dynamic assessment of central corneal inflammatory cells density may provide new insights for management of side effects and, probably, serve as an indicator of miglustat-caused neuropathy's severity following long-term therapy.

To date, very often ophthalmologic findings and the relationship between ocular and extraocular symptoms are neglected in the global evaluation of the LSD-patients. Nevertheless, the ocular involvement can alternatively reflect other more generalized defects in LSDs [35]. Keeping in mind the evidence that *in vivo* CLSM allows the early recognition of morphological changes in the cornea during the progression of disease or treatment course, we believe that this technique has the potential to become an additional clinical tool for reliable diagnosis and evaluation of treatment options in NPC1 disorder.

## Materials and Methods

### Animals

All animal procedures used in this study were approved by the Animal Use and Care Committee of the University of Rostock (approval ID: 7221.3-1.1-088/10) and are in accordance with ARVO Statement for the Use of Animals in Ophthalmic and Vision Research. Breeding pairs of BALB/cNctr-Npc1m1N/-J mice were obtained from Jackson Laboratories (Bar Harbor, ME, USA). These mice were bred to produce normal (NPC1^+/+^), heterozygous (NPC1^+/−^) and homozygous affected (NPC1^−/−^) mice. All mouse pups were genotyped by using a polymerase chain reaction (PCR) assay.

Twenty NPC1^+/+^ and 20 NPC1^−/−^ mice were involved in the treatment study. Fourteen mice from each group were treated by combined SRT and BPT- Cyclo/ALLO/miglustat. Six NPC1^+/+^ and 6 NPC1^−/−^ underwent sham treatment.

At the age of 65 days *in vivo* confocal laser-scanning microscopy (CLSM) was performed on both eyes of each animal. Thereafter, the animals were sacrificed by an overdose of pentobarbital, and the excised eyes were further processed for electron microscopy or High Performance Liquid Chromatography/Mass spectroscopy (HPLC/MS).

### Drug administration

Starting at postnatal day 7 (P7) and weekly thereafter, mice were injected with Cyclodextrin/Allopregnanolone (25 mg/kg ALLO dissolved in 40% CYCLO) (both from Sigma-Aldrich, Munich, Germany). Additionally, the mice were daily injected with miglustat, dissolved in NaCl, 300 mg/kg (miglustat was a kind gift from Actelion Pharmaceuticals, Freiburg, Germany) at P10 until P23. Starting at P23 and until termination of experiments the mice were fed powdered chow with daily addition of miglustat (1200 mg/kg,). In the sham-treated group (both, NPC1^+/+^ and NPC1^−/−^) the mice were injected with 50 µl 0.9% NaCl at P7 and thereafter daily, starting at P10 until P23. Like therapy group the sham-treated mice were fed powdered chow starting at P23.

### In vivo confocal laser-scanning microscopy (CLSM)

HRT II/RCM (Heidelberg Engineering GmbH, Heidelberg, Germany) adapted for veterinary use, was used to examine the corneas of the mice. For *in vivo* CLSM examination mice were anesthetized with 2 mg/kg body weight of xylazine hydrochloride (Rompun; Bayer HealthCare, Leverkusen, Germany) and 50 mg/kg ketamine hydrochloride (Bela-Pharm GmbH & Co KG, Vechta, Germany).

The laser source was a diode laser with a wavelength of 670 nm, and the objective of the microscope was a water immersion lens with magnification ×63 (Zeiss, Hamburg, Germany). The objective lens was fitted with a sterile polymethyle methacrylate cap. During examination a drop of carbomer gel (Vidisic™, Bausch & Lomb/Mann Pharma, Berlin, Germany) was applied as a coupling medium between the lens cap and the cornea. The field of view was 384×384 pixels (image size 300×300 µm) and the focal plane could be moved through the entire cornea. Each cornea was scanned in z-direction 6 times for collecting image stacks, in different x-y positions in the area, including 2 mm of central cornea. Oblique sections of the cornea were obtained by controlling manually the x–y position and the depth of the optical section. Each scan took approximately 20 seconds, and the overall examination, including animal positioning and focus adjustment time, took 10 minutes per cornea.

For quantification of dendritic cells (DCs) HRT-associated cell count software was used. For every cornea each DC was marked once in a predetermined area. DCs touching the border lines were counted only along the upper and right border. DCs touching the left and lower border, were not counted. Statistical analysis was conducted using one side t-test for two independent samples.

### Electron Microscopy

Corneas from following mice were used for transmission electron microscopy: NPC1^+/+^ (untreated control, n = 1), NPC1^+/+^ (treated, n = 1), NPC1^−/−^ treated with no hyperreflective depositions in *in vivo* CLSM (n = 1) and NPC1^−/−^ treated with hyperreflective depositions in *in vivo* CLSM (n = 1). Both corneas of each mouse were excised and fixed in 3.7% paraformaldehyde (PFA) for 1 hour, followed by postfixation in 0.1 M cacodylate buffer containing 2.5% glutaraldehyde for at least 24 hours at 4°C. Thereafter, the specimens were osmicated, washed, block contrasted with 2% aqueous uranyl acetate, dehydrated through a graded series of ethanol, and embedded in Epon 812 (Plano GmbH, Marburg, Germany). Ultrathin sections (about 70 nm) were mounted on pioloform-coated slot copper grids and contrasted with uranyl acetate (8 minutes) followed by lead citrate (2 min). The specimens were examined with a Zeiss EM 902 transmission electron microscope (Zeiss, Oberkochen, Germany) at 80 kV. Photographs were taken using a CCD camera, scanned and adjusted using Photoshop CS2 software.

### High Performance Lipid Chromatography/Mass spectroscopy (HPLC/MS)

For lipid analysis both corneas from randomly selected NPC1^+/+^ (n = 4) and NPC1^−/−^ were harvested and weighed. The samples were immediately frozen in liquid nitrogen and stored at −80°C until further analysis. Both corneas for each animal were pooled to form one sample. Total lipid extractions were obtained by using ultrasonic tissue disintegration for 2 minutes. Total lipids were extracted into ethanolic solution of internal standard (deuterated). Thereafter HPLC/MS was carried out on a C8 column (ACE 3C8, 50×2.1 mm) for determination of free cholesterol and 2 different isoforms (C20-0 and C18-0) of disialotetrahexosylganglioside 2 (GM2). The values were expressed as mg per g wet weight of tissue (mg/g). For statistical analysis non-parametric U-test was performed.

## References

[1] Hers HG (1965). Inborn lysosomal diseases.. Gastroenterology.

[2] Poupetová H, Ledvinová J, Berná L, Dvoráková L, Kozich V (2010). The birth prevalence of lysosomal storage disorders in the Czech Republic: comparison with data in different populations.. J Inherit Metab Dis.

[3] Vanier MT, Millat G (2003). Niemann-Pick disease type C.. Clin Genet.

[4] Millat G, Marçais C, Rafi MA, Yamamoto T, Morris JA (1999). Niemann-Pick C1 disease: the I1061T substitution is a frequent mutant allele in patients of Western European descent and correlates with a classic juvenile phenotype.. Am J Hum Genet.

[5] Liscum L, Klansek JJ (1998). Niemann-Pick disease type C.. Curr Opin Lipidol.

[6] Strauss JF, Liu P, Christenson LK, Watari H (2002). Sterols and intracellular vesicular trafficking: lessons from the study of NPC1.. Steroids.

[7] Vanier MT, Wenger DA, Comly ME, Rousson R, Brady RO (1988). Niemann-Pick disease group C: clinical variability and diagnosis based on defective cholesterol esterification. A collaborative study on 70 patients.. Clin Genet.

[8] Vanier MT, Suzuki K (1998). Recent advances in elucidating Niemann-Pick C disease.. Brain Pathol.

[9] Harzer K, Schlote W, Peiffer J, Benz HU, Anzil AP (1978). Neurovisceral lipidosis compatible with Niemann-Pick disease type C: morphological and biochemical studies of a late infantile case and enzyme and lipid assays in a prenatal case of the same family.. Acta Neuropathol.

[10] Elleder M, Jirasek A, Smid F, Ledvinova J, Besley GT (1985). Niemann-Pick disease type C: study on the nature of the cerebral storage process.. Acta Neuropathol Berl.

[11] Suzuki K, Parker CC, Pentchev PG, Katz D, Ghetti B (1995). Neurofibrillary tangles in Niemann-Pick disease type C.. Acta Neuropathol.

[12] Iturriaga C, Pineda M, Fernández-Valero EM, Vanier MT, Coll MJ (2006). Niemann-Pick C disease in Spain: clinical spectrum and development of a disability scale.. J Neurol Sci.

[13] Vanier MT (2010). Niemann-Pick disease type C.. Orphanet J Rare Dis.

[14] Altmann SW, Davis HR, Zhu LJ, Yao X, Hoos LM (2004). Niemann-Pick C1 Like 1 protein is critical for intestinal cholesterol absorption.. Science.

[15] Zhang M, Li J, Chakrabarty P, Bu B, Vincent I (2004). Cyclin-dependent kinase inhibitors attenuate protein hyperphosphorylation, cytoskeletal lesion formation, and motor defects in Niemann-Pick Type C mice.. Am J Pathol.

[16] Zhang M, Hallows JL, Wang X, Bu B, Wang W (2008). Mitogen-activated protein kinase activity may not be necessary for the neuropathology of Niemann-Pick type C mice.. J Neurochem.

[17] Zervas M, Somers KL, Thrall MA, Walkley SU (2001). Critical role for glycosphingolipids in Niemann-Pick disease type C.. Curr Biol.

[18] Lachmann RH, te Vruchte D, Lloyd-Evans E, Reinkensmeier G, Sillence DJ (2004). Treatment with miglustat reverses the lipid-trafficking defect in Niemann-Pick disease type C.. Neurobiol Dis.

[19] Chien YH, Lee NC, Tsai LK, Huang AC, Peng SF (2007). Treatment of Niemann-Pick disease type C in two children with miglustat: initial responses and maintenance of effects over 1 year.. J Inherit Metab Dis.

[20] Patterson MC, Vecchio D, Prady H, Abel L, Wraith JE (2007). Miglustat for treatment of Niemann-Pick C disease: a randomised controlled study.. Lancet Neurol.

[21] Pineda M, Wraith JE, Mengel E, Sedel F, Hwu WL (2009). Miglustat in patients with Niemann-Pick disease Type C (NP-C): a multicenter observational retrospective cohort study.. Mol Genet Metab.

[22] Wraith JE, Vecchio D, Jacklin E, Abel L, Chadha-Boreham H (2010). Miglustat in adult and juvenile patients with Niemann-Pick disease type C: long-term data from a clinical trial.. Mol Genet Metab.

[23] Griffin LD, Gong W, Verot L, Mellon SH (2004). Niemann-Pick type C disease involves disrupted neurosteroidogenesis and responds to allopregnanolone.. Nat Med.

[24] Langmade SJ, Gale SE, Frolov A, Mohri I, Suzuki K (2006). Pregnane X receptor (PXR) activation: a mechanism for neuroprotection in a mouse model of Niemann-Pick C disease.. Prot Natl Acad Sci USA.

[25] Davidson CD, Ali NF, Micsenyi MC, Stephney G, Renault S (2009). Chronic cyclodextrin treatment of murine Niemann-Pick C disease ameliorates neuronal cholesterol and glycosphingolipid storage and disease progression.. PLoS One.

[26] Hovakimyan M, Stachs O, Reichard M, Mascher H, Lukas J (2011). Morphological alterations of the cornea in the mouse model of niemann-pick disease type c1.. Cornea.

[27] Loftus SK, Morris JA, Carstea ED, Gu JZ, Cummings C (1997). Murine model of Niemann-Pick C disease: mutation in a cholesterol homeostasis gene.. Science.

[28] Nunes A, Pressey SN, Cooper JD, Soriano S (2011). Loss of amyloid precursor protein in a mouse model of Niemann-Pick type C disease exacerbates its phenotype and disrupts tau homeostasis.. Neurobiol Dis.

[29] Morris MD, Bhuvaneswaran C, Shio H, Fowler S (1982). Lysosome lipid storage disorder in NCTR-BALB/c mice. I. Description of the disease and genetics.. Am J Pathol.

[30] Xie C, Turley SD, Dietschy JM (1999). Cholesterol accumulation in tissues of the Niemann-pick type C mouse is determined by the rate of lipoprotein-cholesterol uptake through the coated-pit pathway in each organ.. Proc Natl Acad Sci U S A.

[31] Sawamura N, Gong JS, Garver WS, Heidenreich RA, Ninomiya H (2001). Site-specific phosphorylation of tau accompanied by activation of mitogen-activated protein kinase (MAPK) in brains of Niemann-Pick type C mice.. J Biol Chem.

[32] Claudepierre T, Paques M, Simonutti M, Buard I, Sahel J (2009). Lack of Niemann-Pick type C1 induces age-related degeneration in the mouse retina.. Mol Cell Neurosci.

[33] Abel LA, Walterfang M, Fietz M, Bowman EA, Velakoulis D (2009). Saccades in adult Niemann-Pick disease type C reflect frontal, brainstem, and biochemical deficits.. Neurology.

[34] Brady RO (1978). Ophthalmologic aspects of lipid storage diseases.. Ophthalmology.

[35] Biswas J, Nandi K, Sridharan S, Ranjan P (2008). Ocular manifestation of storage diseases.. Curr Opin Ophthalmol.

[36] Palmer M, Green WR, Maumenee IH, Valle DL, Singer HS (1985). Niemann-Pick disease–type C. Ocular histopathologic and electron microscopic studies.. Arch Ophthalmol.

[37] Emery JM, Green WR, Huff DS, Sloan HR (1972). Niemann-Pick disease (type C). Histopathology and ultrastructure.. Am J Ophthalmol.

[38] Herrmann WA, von Mohrenfels CW, Lohmann CP (2004). Confocal microscopy and corneal sensitivity in a patient with corneal manifestations of Tangier disease.. Cornea.

[39] Falke K, Büttner A, Schittkowski M, Stachs O, Kraak R (2009). The microstructure of cornea verticillata in Fabry disease and amiodarone-induced keratopathy: a confocal laser-scanning microscopy study.. Graefes Arch Clin Exp Ophthalmol.

[40] Tavares R, Coelho D, Macário MC, Torres A, Quadrado MJ (2009). Evaluation of treatment with cysteamine eyedrops for cystinosis with confocal microscopy.. Cornea.

[41] Maegawa GH, van Giersbergen PL, Yang S, Banwell B, Morgan CP (2009). Pharmacokinetics, safety and tolerability of miglustat in the treatment of pediatric patients with GM2 gangliosidosis.. Mol Genet Metab.

[42] Tavakoli M, Boulton AJ, Efron N, Malik RA (2010). Increased Langerhan cell density and corneal nerve damage in diabetic patients: role of immune mechanisms in human diabetic neuropathy.. Cont Lens Anterior Eye.

